# Acromiohumeral Distance as a Diagnostic and Prognostic Biomarker for Shoulder Disorders: A Systematic Review—Acromiohumeral Distance and Shoulder Disorders

**DOI:** 10.3390/jfmk10040478

**Published:** 2025-12-15

**Authors:** Luis Alfonso Arráez-Aybar, Carlos Miquel García-de-Pereda-Notario, Luis Palomeque-Del-Cerro, Juan José Montoya-Miñano

**Affiliations:** 1 Department of Anatomy and Embryology, School of Medicine, Complutense University of Madrid, 28040 Madrid, Spain; arraezla@med.ucm.es; 2 UCM Research Group No. 920202, School of Medicine, Complutense University of Madrid, 28040 Madrid, Spain; 3 Department of Physiotherapy, School of Nursing and Physiotherapy “Salus Infirmorum”, Pontifical University of Salamanca, 28015 Madrid, Spain; lpalomequede@upsa.es; 4 Escuela de Osteopatía de Madrid, 28033 Madrid, Spain; 5 Department of Radiology and Rehabilitation, School of Medicine, Complutense University of Madrid, 28040 Madrid, Spain; jjmontoy@ucm.es

**Keywords:** acromiohumeral distance, shoulder pain, rotator cuff tear, ultrasound, imaging, diagnostic accuracy, subacromial space

## Abstract

**Objectives:** The acromiohumeral distance (AHD) is widely used to evaluate subacromial pathology, particularly rotator cuff–related disorders. However, substantial heterogeneity exists across studies in imaging protocols, measurement definitions, and diagnostic thresholds. This systematic review aimed to synthesize current evidence on AHD measurement methods, assess reliability and diagnostic performance across imaging modalities, and examine the clinical relevance of AHD as both a structural and functional biomarker. **Methods:** A systematic search of PubMed, Web of Science, and SciELO (January 2006–May 2025) was conducted following PRISMA 2020. Eligible studies reported quantitative AHD measurements using ultrasound, MRI, or radiography in adults. Two reviewers independently conducted screening, extraction, and QUADAS-2 assessments. Due to heterogeneity, results were narratively synthesized. **Results:** Twenty-nine studies met the inclusion criteria. Definitions of AHD and imaging procedures varied substantially. Ultrasound showed the most consistent intra- and inter-observer reliability, whereas MRI and radiography demonstrated greater protocol-dependent variability. Reduced AHD values were frequently associated with full-thickness rotator cuff tears, while larger values typically characterized asymptomatic individuals. Several studies also reported reductions in AHD during arm elevation, supporting its interpretation as a functional parameter influenced by scapular motion and neuromuscular control. **Conclusions:** AHD is a reliable and clinically informative measure when acquired using standardized protocols, with Ultrasound demonstrating the highest reproducibility. Its sensitivity to positional and dynamic factors supports its role as both a structural and functional biomarker. Further research should prioritize standardized imaging procedures, dynamic assessment methods, and evaluation of emerging technologies to improve the diagnostic and prognostic value of AHD.

## 1. Introduction

Shoulder pain is among the most common musculoskeletal complaints, affecting up to one-third of the general population at some point and often resulting in functional limitations and reduced quality of life [1,2,3]. A critical anatomical structure in this context is the subacromial space, radiologically represented by the acromiohumeral distance (AHD), defined as the vertical distance between the inferior acromial margin and the humeral head [4,5].

Multiple studies have demonstrated that reduced AHD values are significantly associated with subacromial pain syndrome (SAPS) and rotator cuff tears (RCTs), particularly those involving the supraspinatus tendon [6,7]. Although several studies have reported that an AHD ≤ 6 mm is often associated with high specificity—and occasionally high sensitivity—for detecting full-thickness rotator cuff tears [8,9], these diagnostic values vary across imaging modalities and study populations. Collectively, these associations have positioned AHD as a structural biomarker with diagnostic and prognostic relevance, especially in pre- and postoperative assessments [3,10,11].

Although clinical examination remains essential, physical orthopedic tests alone often lack the sensitivity and specificity to accurately detect RCTs or to differentiate structural from functional subacromial pathology [3,12]. Imaging techniques—particularly ultrasound (US), magnetic resonance imaging (MRI), and conventional radiography (X-ray)—enable direct, quantitative evaluation of AHD, complementing clinical assessment [13,14]. Each modality has specific advantages and limitations: radiography is affordable and widely available but sensitive to arm position and projection angle and lacks soft-tissue detail [8,15]; MRI provides high-resolution soft-tissue imaging and is often considered the reference standard for tendon integrity, although it is less applicable for dynamic assessment and more costly [9,11]; and US has gained recognition for its dynamic capability, low cost, portability, and excellent intra- and inter-observer reliability, particularly when performed by trained clinicians [16,17,18,19].

Clinical implementation of AHD measurement remains inconsistent due to the absence of universal protocols, variable cut-offs, and heterogeneity across populations and imaging settings. Although multiple studies have demonstrated the diagnostic utility of AHD, significant methodological limitations persist in the current literature. These include inconsistencies in technical variables such as arm positioning during image acquisition (e.g., variable degrees of abduction or rotation), scapular stabilization and orientation, probe angulation and pressure during ultrasound assessment, and variability in radiographic projection angles. These factors are known to modify the measured distance and contribute to between-study heterogeneity [13,15,16,20]. However, existing studies and prior reviews show substantial methodological heterogeneity, with inconsistent imaging protocols, variable arm positions, and differing diagnostic thresholds for defining pathological AHD [13,16,20]. Moreover, no recent synthesis has integrated reliability, diagnostic accuracy, and dynamic assessment across imaging modalities. These shortcomings highlight the need for an updated and comprehensive systematic review. Accordingly, the aims of this systematic review were to: (i) synthesize current evidence on AHD measurement methods across imaging modalities; (ii) determine normative values and diagnostic performance parameters of AHD, including pathological thresholds and measurement reliability; and (iii) explore clinical and surgical implications of AHD measurement in the assessment of shoulder pain.

## 2. Materials and Methods

### 2.1. Study Design and Reporting Standards

This systematic review was conducted in accordance with PRISMA 2020 guidelines [21]. The review protocol was structured following PRISMA recommendations for transparency in eligibility criteria, search strategy, study selection, data extraction, and risk-of-bias assessment. No automation tools or machine-learning–based filters were used during any stage of screening or selection; all steps were completed manually.

### 2.2. Search Strategy

A comprehensive search of MEDLINE (via PubMed), SciELO and Web of Science was performed, covering the period from January 2006 to May 2025. The cutoff of January 2006 was selected because standardized ultrasound protocols for AHD measurement began to emerge after 2005, including consistent probe orientation, arm positioning, and measurement conventions. Earlier studies used heterogeneous definitions of AHD and lacked reproducible methodology; therefore, restricting inclusion to 2006 onward improved comparability.

Search terms combined controlled vocabulary and keywords related to AHD, subacromial space, imaging modalities (US, MRI, X-ray), rotator cuff pathology, and shoulder pain. Full database-specific search strings for transparency and reproducibility are provided in Appendix A, as required by PRISMA 2020.

### 2.3. Eligibility Criteria

Eligible studies were those that reported quantitative measurements of the AHD obtained through US, MRI, or X-ray in adult participants (≥18 years), including both asymptomatic individuals and patients with shoulder pathology such as subacromial pain syndrome, tendinopathy, and partial- or full-thickness rotator cuff tears. Postoperative cases following rotator cuff repair and athletic or overhead populations were also eligible when quantitative AHD measurements were available. Studies were included regardless of clinical setting, provided that AHD was measured using clearly defined anatomical landmarks and acceptable imaging protocols, specifically defined as the shortest perpendicular distance between the inferior acromial surface and the humeral head. Exclusion criteria comprised cadaveric or animal studies, investigations lacking quantitative AHD values, interventional studies without baseline AHD measurement, conference abstracts without full datasets, and review articles or commentaries. Systematic reviews and meta-analyses were screened solely to identify additional primary studies; no diagnostic or reliability data were extracted from secondary sources to avoid duplication and maintain methodological integrity.

### 2.4. Study Selection

**Figure 1:**
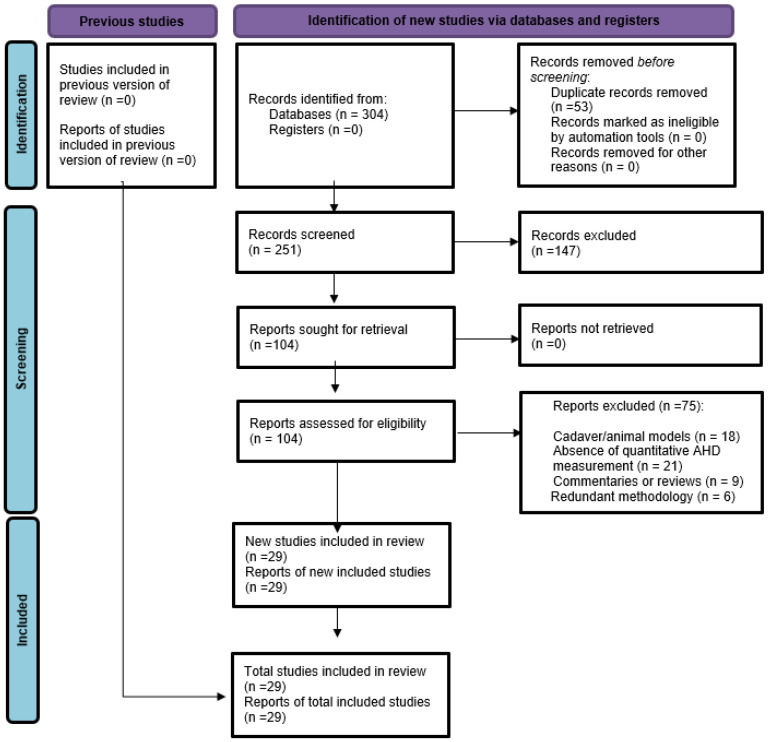
PRISMA Flow Diagram. PRISMA flow diagram illustrating study selection.

Following the database search, duplicate records were removed manually. Title and abstract screening were performed independently by two reviewers using predefined inclusion and exclusion criteria (LAAA and CMGPN). Full-text assessment of potentially eligible studies was also conducted independently by two reviewers (LAAA and LPdC). Any disagreements were resolved through discussion, and when necessary, consultation with a third reviewer (JJMM). A PRISMA flow diagram summarizing identification, screening, eligibility, and inclusion stages is presented in Figure 1.

### 2.5. Data Extraction

Data extraction was performed independently by two reviewers using a standardized template. Extracted variables included study design, population characteristics, imaging modality, measurement protocol, AHD values, reliability measures, including Intraclass Correlation Coefficient (ICC), diagnostic accuracy parameters (sensitivity, specificity), and methodological features.

The data extraction form was piloted independently by two reviewers using a subset of three studies to ensure consistency and completeness. Missing or ambiguous data were resolved by contacting study authors when possible or were otherwise documented qualitatively.

Disagreements were resolved through discussion and consensus between reviewers, with the involvement of a third reviewer when required.

All diagnostic and reliability outcomes included in this review were extracted exclusively from primary studies; no data were taken from systematic reviews or meta-analyses to avoid duplication and maintain methodological integrity.

Inter-rater agreement statistics (e.g., Cohen’s κ) were not calculated because disagreements during screening and extraction were resolved by discussion and consensus between the two reviewers, with the involvement of a third reviewer when necessary.

### 2.6. Level of Evidence

Each included study was classified according to the Oxford Centre for Evidence-Based Medicine (OCEBM) Levels of Evidence [22], ranging from Level I (high-quality systematic reviews or cohort studies with external validation) to Level III (non-randomized, observational, or cross-sectional studies). No Level IV or V studies were included.

### 2.7. Methodological Quality Assessment (QUADAS-2)

Risk of bias was assessed using QUADAS-2 [23], following its four domains: patient selection, index test, reference standard, and flow/timing. The assessment was conducted independently by two reviewers (LAAA and JJMM). A third author (LPC) arbitrated disagreements. QUADAS-2 ratings were used to inform the interpretation of diagnostic accuracy findings but did not determine study inclusion. All studies meeting eligibility criteria were retained regardless of risk-of-bias level.

Overall applicability concerns and risk-of-bias results are summarized below.

### 2.8. Data Synthesis

**Figure 2:**
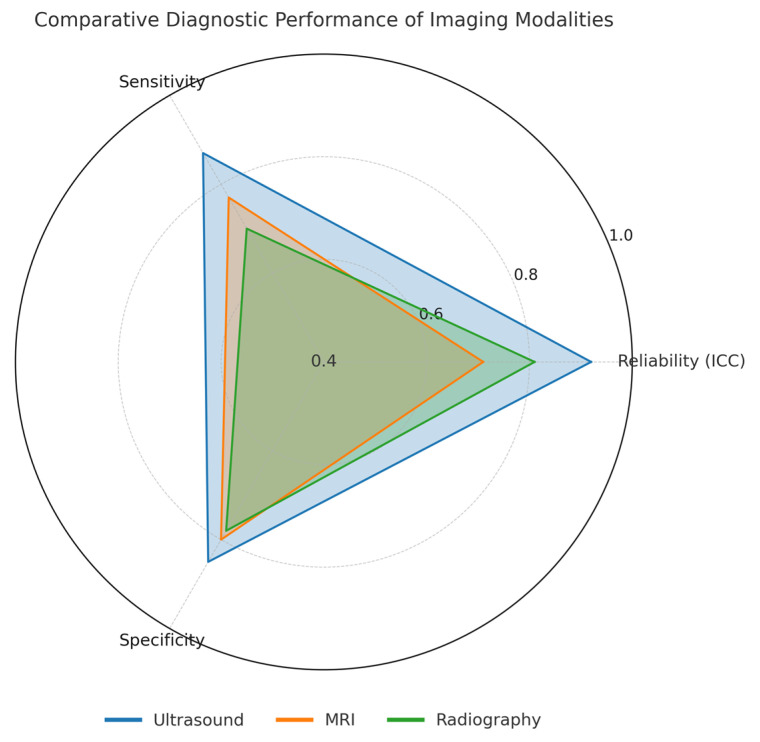
Comparative Diagnostic Performance.

Given the heterogeneity of imaging protocols, measurement techniques, patient postures, acromial morphologies, and diagnostic thresholds, quantitative pooling was not feasible. Therefore, a structured narrative synthesis was performed, integrating diagnostic performance, reliability, and clinical implications across modalities. Summary values for ICC, sensitivity, and specificity are presented in below and visually synthesized in Figure 2.

## 3. Results

### 3.1. Study Selection

The database search initially yielded 304 records. After removing duplicates (n = 53), 251 titles and abstracts were screened, leading to the exclusion of 147 studies. Of 104 full-text articles assessed for eligibility, 54 were excluded for not meeting inclusion criteria. Reasons for exclusion included: absence of quantitative AHD measurements, cadaveric or animal study design, review or commentary format, and redundancy due to overlapping methodologies. In total, 29 studies were included in the qualitative synthesis (Figure 1, PRISMA flow diagram). One study was excluded from the QUADAS-2 analysis (Section 3.3).

### 3.2. Characteristics of Included Studies

Populations included asymptomatic individuals, patients with SAPS, RCTs, spinal cord injury, and postoperative cases. Designs comprised 6 systematic reviews or prospective cohorts with clear diagnostic criteria (Level I), 18 observational comparative studies (Level II), and 5 cross-sectional/exploratory studies (Level III), per OCEBM criteria [22]. Summary details of imaging techniques, study designs, and levels of evidence are provided in Table 1. US was the most frequently used modality (n = 16, 48%), followed by MRI (n = 9, 31%) and radiography (n = 5, 17%); seven studies used more than one imaging modality. Six studies applied dynamic or loaded protocols [1,2,18,19,20,24]. Twenty-three studies reported intra-observer ICCs and 16 reported inter-observer ICCs.

### 3.3. Methodological Quality (QUADAS-2)

Of the 29 studies included in the qualitative synthesis, one [32] was excluded from QUADAS-2 because it did not report direct AHD measurements. Thus, 28 studies were assessed (Table 2). Overall, most studies demonstrated low risk of bias and high applicability across domains. Across the included studies, patient selection and index test domains were consistently rated as low risk. This reflects strong methodological reporting within these domains, although the uniformity of these ratings should not be interpreted as implying methodological perfection, but rather appropriate alignment with QUADAS-2 criteria. Flow and timing were also rated as low risk in all studies. For the reference standard domain, 18 studies (64.3%) were rated as low risk, 3 (10.7%) as high risk, and 7 (25%) as unclear due to insufficient information. Applicability concerns were uniformly low for patient selection and index test, whereas the reference standard domain showed greater variability (64.3% low, 10.7% high, and 25% unclear).

**Table 2:** QUADAS-2 summary across domains.

Authors	1	2	3	4	A	B	C	Comments
Bahtiyar [25]	Low	Low	High	Low	Low	Low	High	Clear inclusion/exclusion
Boulanger [18]	Low	Low	Low	Low	Low	Low	Low	Well-controlled comparative US-MRI study with standardized protocols and blinded assessment.
Cavaggion [19]	Low	Low	Unclear	Low	Low	Low	Unclear	Focused on inter-rater reliability in symptomatic vs. asymptomatic population using ultrasound.
Dede [26]	Low	Low	High	Low	Low	Low	High	Wireless vs. standard US without independent gold standard comparison.
Dede [27]	Low	Low	Low	Low	Low	Low	Low	MRI-based reliability study with consistent raters and measurements on same day.
Deger [4]	Low	Low	Low	Low	Low	Low	Low	Compared AHD measurements across imaging modalities with good internal consistency.
Gruber [5]	Low	Low	Unclear	Low	Low	Low	Unclear	Reliability study of radiographic AHD with limited reference standard data
Kholinne [10]	Low	Low	Low	Low	Low	Low	Low	Meta-analysis and systematic review with consistent inclusion/exclusion and robust methods.
Kizilay [28]	Low	Low	Unclear	Low	Low	Low	Unclear	Volumetric MRI analysis without gold standard comparison.
Kocadal [29]	Low	Low	Unclear	Low	Low	Low	Unclear	MRI-based volume estimation of subacromial space
Kozono [20]	Low	Low	Low	Low	Low	Low	Low	Dynamic AHD measured via fluoroscopy
Leong [30]	Low	Low	Unclear	Low	Low	Low	Unclear	Focused on reliability of US
Lin [1]	Low	Low	Unclear	Low	Low	Low	Unclear	Ultrasound-based AHD in spinal cord injury
McCreesh [31]	Low	Low	Low	Low	Low	Low	Low	Systematic review with multiple studies on AHD and clear methodological quality.
McCreesh [16]	Low	Low	Unclear	Low	Low	Low	Unclear	Reliability of US in tendinopathy
Michener [13]	Low	Low	High	Low	Low	Low	High	Reliability of US for supraspinatus tendon thickness and AHD
Navarro-Ledesma [2]	Low	Low	Low	Low	Low	Low	Low	Case–control design with adequate blinding and standard protocols for US.
Park [11]	Low	Low	Low	Low	Low	Low	Low	Systematic review and meta-analysis
Pieters [3]	Low	Low	Low	Low	Low	Low	Low	Systematic review with high-quality synthesis of conservative therapy impact on AHD.
Rentz [17]	Low	Low	Low	Low	Low	Low	Low	Methodological study with blinded raters and reliability statistics (ICC) for US measurements.
Sakdapanichkul [33]	Low	Low	Low	Low	Low	Low	Low	Proposes a novel ratio (AHD/Glenoid width) with strong design and radiographic reliability.
Sanguanjit [15]	Low	Low	Low	Low	Low	Low	Low	Comparison of upright vs. supine AHD
Saupe [8]	Low	Low	Low	Low	Low	Low	Low	Large sample MRI-based study correlating AHD with cuff tears
Sürücü [6]	Low	Low	Low	Low	Low	Low	Low	MRI and radiography bilateral comparison
Wynne [24]	Low	Low	Low	Low	Low	Low	Low	US evaluation of GH mobilization effects on AHD
Xu [7]	Low	Low	Low	Low	Low	Low	Low	US based correlation of AHD and supraspinatus tear severity
Xu [9]	Low	Low	Low	Low	Low	Low	Low	Retrospective case–control study using MRI with validated measures of AHD.
Yuan [14]	Low	Low	Low	Low	Low	Low	Low	US-based reliability assessment in healthy population

Domains of potential bias: 1: Patient selection bias; 2: Index test bias; 3: Reference standard bias; 4: Flow and time bias. Domains of applicability: A: Patient selection; B: Index test; C: Reference standard. Low = low risk of bias; High = high risk of bias; Unclear = insufficient information to judge.

### 3.4. Measurement Techniques and Imaging Modalities

In the studies included, the AHD was consistently defined as the shortest perpendicular distance between the inferior acromial surface and the humeral head. The Acriohumeral interval-Glenoid Ratio (AHIGR) was reported as a radiographic index expressing the relationship between AHD and the glenoid width (AHD/Glenoid Width) to normalize measurements across individuals. ‘Dynamic AHD’ referred to measurements obtained during arm elevation or movement, in contrast to static assessments performed at rest.

AHD was measured using ultrasound, MRI, and X-ray across the included studies. US was the most frequently used modality, and several investigations incorporated positional or dynamic protocols. The AHIGR was reported in two studies as an alternative radiographic index. Arm position, probe orientation, and patient posture varied substantially across studies.

### 3.5. Reliability and Diagnostic Accuracy

Across the 29 included studies, 15 reported ICC-based reliability metrics, of which 12 provided intra-observer ICCs and 12 reported inter-observer ICCs. Three studies reported diagnostic sensitivity and specificity. Normative AHD values were available in 11 studies, whereas 7 studies reported pathological thresholds or diagnostic cut-offs. Dynamic AHD measurements during shoulder movement were described in 6 studies. Regarding imaging modalities, 13 studies used ultrasound, 10 used MRI, and 9 used radiography, with several investigations employing more than one technique.

Reliability values showed wide variability across protocols, with intra-observer ICCs ranging from 0.75 to 0.98 and inter-observer ICCs from 0.52 to 0.97, reflecting differences in examiner experience, arm positioning, and landmark selection. Sensitivity values in the diagnostic-accuracy studies ranged from 22% to 75%, while specificity ranged from 67% to 100%. Normative AHD measurements in asymptomatic individuals commonly ranged from 8 to 13 mm at rest and decreased to 5–7 mm during elevation, whereas pathological values consistently fell at or below 6–7 mm across modalities. Substantial heterogeneity was observed across studies in arm position (0–90° abduction), plane of measurement, imaging modality, examiner expertise, and diagnostic thresholds.

US generally demonstrated excellent reliability across studies, with intra- and inter-observer ICC values typically ranging from 0.88 to 0.98 and reaching up to 0.996 in standardized protocols. MRI showed more variable reliability, with ICC values between 0.57 and 0.96, reflecting differences in acquisition protocols, image interpretation, and the use of static versus volumetric measurements. Radiography demonstrated moderate reliability, with ICC values ranging from 0.75 to 0.91 depending on projection technique and observer experience.

AHD values ≤ 6 mm showed high specificity for full-thickness rotator cuff tears, whereas values of approximately ≥7 mm were most commonly reported as normal in asymptomatic shoulders. These diagnostic thresholds were supported primarily by studies using ultrasound and MRI.

A comparative overview of diagnostic performance and measurement characteristics for each imaging modality is presented in Table 3 and Figure 2.

**Table 3:** Comparative summary of imaging modalities for AHD measurement.

Imaging Modality	Reliability (ICC)	Sensitivity	Specificity	Clinical Strengths/Limitations
Ultrasound (US)	0.85–0.98 (excellent)	>85%	80–90%	Dynamic, portable, low cost; operator-dependent; requires standardization.
Magnetic Resonance Imaging (MRI)	0.57–0.85 (variable)	70–85% (varies)	75–85%	Detailed anatomy; postoperative evaluation; higher cost; limited dynamic assessment.
Radiography (X-ray)	0.77–0.85 (moderate)	<60% (partial tears) ~80% (full-thickness)	~78% (complete tears)	Accessible, inexpensive; projection artifacts; low sensitivity for partial tears.

Radar chart illustrating mean ICC, sensitivity, and specificity values for US, MRI, and X-ray. Values represent averaged estimates derived from the corresponding ranges reported in Table 3. Label positions were adjusted to improve readability.

### 3.6. Clinical and Surgical Findings

Several studies explored clinical or surgical correlates of AHD. Lower AHD values were associated with the presence and severity of rotator cuff tears, particularly full-thickness lesions. Dynamic or positional assessments demonstrated reductions in AHD during arm elevation in individuals with subacromial pain or rotator cuff pathology. Three studies reported that AHD values ≤ 6–7 mm were strongly associated with massive or full-thickness tears, whereas normative ranges were generally wider (8–13 mm). Pre- and postoperative comparisons showed that surgical repair could increase AHD in some cases, although results varied across procedures and imaging modalities. The AHIGR demonstrated high specificity for massive rotator cuff tears in radiographic evaluations.

## 4. Discussion

This systematic review synthesizes current evidence on the clinical and biomechanical significance of the AHD across imaging modalities. Consistent patterns emerged across studies: reduced AHD values were strongly associated with subacromial pathologies—particularly full-thickness or massive RCTs [7,8]—whereas larger distances characterized asymptomatic individuals [2,5]. US consistently demonstrated the most reproducible AHD measurements, while MRI and radiography showed greater variability due to differences in acquisition protocols, arm positioning, and image interpretation [16,20].

Although traditionally interpreted as a static anatomical interval, multiple studies indicate that AHD behaves as a dynamic parameter influenced by scapular kinematics, humeral head translation, and neuromuscular control [1,2,19,20,24]. This pattern supports the concept of AHD as a functional biomarker reflecting the interaction between structural integrity and the real-time behavior of the subacromial space. Reductions in AHD during active elevation, and its responsiveness to clinical or postoperative change, further reinforce this functional interpretation [24].

US-based assessments repeatedly showed high reproducibility across observers, likely due to direct visualization of the supraspinatus footprint and consistent measurement protocols [14,16]. MRI, while superior for structural characterization, demonstrated protocol-dependent variability associated with positioning, rotation, and plane selection [9,15,27]. Radiography, although specific for advanced tears, remained sensitive to projection angle and posture [4,8]. These observations highlight how technical factors influence AHD measurement and reinforce the need for standardized imaging approaches.

Diagnostic thresholds across modalities converged around pathological cut-offs ≤ 6–7 mm, a finding supported by MRI and radiographic literature [6,8,33]. Normative AHD values in asymptomatic samples commonly ranged higher, typically between 9 and 12 mm, and consistently decreased with elevation or functional loading—highlighting the need to interpret measurements according to arm position and functional demands [2,14,19,34]. Pre- and postoperative comparisons further suggest that surgical repair may increase AHD in some cases, although outcomes vary across procedures and imaging modalities [10,28]. Collectively, these clinical applications of AHD measurement align with current shoulder management guidelines from the American Academy of Orthopaedic Surgeons [35] and the American Physical Therapy Association [36], which emphasize the integration of imaging findings with functional assessment and evidence-based rehabilitation strategies.

Emerging imaging technologies offer potential solutions to longstanding methodological challenges. Portable ultrasound systems may reduce setting-related variability and improve feasibility in primary care or athletic environments [26]. AI-assisted image analysis can minimize operator dependence by automating landmark detection and measurement [37,38,39]. Three-dimensional ultrasound and volumetric reconstruction methods address inconsistencies in image plane selection, while augmented-reality guidance and tele-ultrasound platforms may improve training and standardization [40,41]. Collectively, these innovations directly target the sources of heterogeneity identified in the included studies—arm positioning, plane selection, and examiner experience.

Overall, the findings of this review demonstrate that AHD represents not only an anatomical measurement, but a sensitive functional marker influenced by posture, muscle activation, and shoulder biomechanics. Integrating static and dynamic AHD assessments may therefore enhance diagnostic precision and support individualized rehabilitation or postoperative monitoring for subacromial shoulder disorders.

This review has several limitations. First, the search was restricted to studies published in English or Spanish, which may have resulted in selection bias. Second, studies lacking quantitative AHD values were excluded, limiting inclusion to investigations with comparable measurement outcomes. Substantial methodological heterogeneity—particularly in arm position, patient posture, and imaging protocols—precluded meta-analysis and required a narrative synthesis. Additionally, the underrepresentation of older adults and elite athletes restricts generalizability to these populations. Finally, variability in reporting standards and inconsistent use of dynamic assessment protocols highlight the need for unified methodological frameworks in future research.

## 5. Conclusions and Future Directions

AHD is a valuable structural and functional biomarker in the assessment of shoulder disorders, particularly those involving rotator cuff pathology and subacromial pain mechanisms. US remains the most reliable modality for AHD measurement, offering dynamic assessment capabilities when standardized protocols are applied. MRI provides detailed soft-tissue characterization, whereas radiography offers a practical screening option in selected clinical contexts.

Across studies, reduced AHD values were associated with rotator cuff tear severity, while higher values characterized asymptomatic individuals. Incorporating both static and dynamic AHD measurements may enhance clinical decision-making by capturing the functional behavior of the subacromial space during movement.

Future research should prioritize standardized imaging protocols across modalities, age- and sex-specific normative values, and clarification of the prognostic role of AHD in rehabilitation and surgical outcomes. Evaluation of emerging AI-assisted, 3D, and tele-ultrasound technologies will be essential to reduce operator dependency, harmonize acquisition parameters, and improve reproducibility across clinical settings.

## Figures and Tables

**Table 1:** Summary of the main included studies by imaging modality, ICC, diagnostic performance, and OCEBM level.

Study	Imaging	ICC Intra	ICC Inter	Sensitivity/Specificity	Evidence Level
Bahtiyar [25]	MRI	0.93–0.96	–	–	III
Boulanger [18]	US vs. MRI	0.83–0.97	0.63–0.74	Validity US–MRI 0.21–0.49	II
Cavaggion [19]	US (dynamic)	–	0.52–0.77	–	II
Dede [26]	US (wireless vs. conventional)	–	0.96–0.97	–	II
Dede [27]	MRI	0.94–0.96	0.75–0.86	–	II
Deger [4]	X-ray vs. US	0.79–0.97	0.82–0.91	US–X-ray Sens 0.68–0.75	II
Gruber [5]	X-ray	–	≤4 mm diff.	Spec >90% (AHD < 6 mm)	I
Kholinne [10]	MRI	–	–	High specificity AHD < 6 mm	II **^††^**
Kizilay [28]	MRI (3D)	0.937	0.906	–	III
Kocadal [29]	MRI (3D)	>0.70	–	–	III
Kozono [20]	Dynamic fluoroscopy (3D–2D registration)	0.90	0.82	–	II
Leong [30]	US	0.92	0.83	–	II
Lin [1]	US (dynamic)	0.94	0.85	–	II
McCreesh [31]	Systematic review—multi-modality	0.96	–	–	I
McCreesh [16]	US	>0.92	>0.90	–	I
Michener [13]	US	0.98	–	–	II
Navarro-Ledesma [2]	US (dynamic)	0.88–0.98	–	–	II
Ogbeivor [32]	US/X-ray (contrast-confirmed)	–	–	Indirect pathological thresholds	I
Park [11]	Meta-analysis	–	–	MD = 0.28 mm (ns)	I
Pieters [3]	Systematic review	–	–	–	I
Rentz [17]	US	0.996	0.959–0.997	–	II
Sakdapanichkul [33]	X-ray (AHI + AHIGR)	0.749–0.923	0.866–0.923	Sens 22–25%; Spec 96–100%	II
Sanguanjit [15]	X-ray vs. MRI	0.668–0.824	0.753–0.824	Sens 28–33%; Spec 100%	II
Saupe [8]	X-ray + MRI	0.77–0.99	–	Sens 90%; Spec 67% (AHD ≤ 7 mm)	II
Sürücü [6]	X-ray + MRI	0.97	0.97	–	III
Wynne [24]	US (dynamic)	0.876–0.963	–	–	II
Xu [7]	US	0.98	–	Correlation with tear severity	III
Xu [9]	MRI	0.906	–	No AHD–pain correlation	II
Yuan [14]	US	0.76–0.79	0.63	–	II

^††^ Although Kholinne et al. [10] is a systematic review, it was classified as Level II due to the lack of primary diagnostic accuracy data specific to AHD. Abbreviations: AHI: Acromiohumeral interval; AHIGR = AHI-Glenoid Ratio (AHD/Glenoid Width); ICC intra/inter= intraclass correlation coefficients intra/ interobserver; MD = Mean Difference; Sens = Sensitivity; Spec = Specificity.

## Data Availability

All extracted and analyzed data are available within the cited studies.

## Abbreviations

AHD: Acromiohumeral Distance; AHD-RC: Acromiohumeral Distance–Rotator Cuff correlation; AHI: Acromiohumeral Interval; CT: Computed Tomography; ICC: Intraclass Correlation Coefficient; MD: Mean Difference; OCEBM: Oxford Centre for Evidence-Based Medicine; RCT: Rotator Cuff Tear; MRI: Magnetic Resonance Imaging; Sens: Sensitivity; SAPS: Subacromial Pain Syndrome; Spec: Specificity; SIS: Subacromial Impingement Syndrome; US: Ultrasound; WUS: Wireless Ultrasound; X-ray: Radiography.

## Appendix A. Full Search Strategies

**PubMed:**

(acromiohumeral distance[MeSH] OR acromiohumeral[Title/Abstract] OR subacromial space[Title/Abstract]) AND (ultrasound OR MRI OR radiography) AND (rotator cuff OR shoulder impingement OR subacromial pain)

**Web of Science:**

TS = (acromiohumeral distance OR subacromial space) AND TS = (ultrasound OR MRI OR radiography) AND TS = (rotator cuff OR impingement OR shoulder pain)

**SciELO:**

(acromiohumeral OR subacromial) AND (ultrasonido OR resonancia magnética OR radiografía)
